# Data of infrared vibration spectroscopy of cyclotriphosphates

**DOI:** 10.1016/j.dib.2019.104075

**Published:** 2019-05-31

**Authors:** Soufiane Zerraf, Malika Tridane, Said Belaaouad

**Affiliations:** a Laboratory of Physical Chemistry of Materials LCPM, Faculty of Sciences Ben M'sik, Hassan II University of Casablanca, B.P. 7955, Bd Cdt Driss El Harti, Morocco; b Regional Center for Education and Training Occupations Casablanca Anfa, Bd Bir Anzarane Casablanca, Morocco

**Keywords:** Infrared, Raman, Vibration spectroscopy, Cyclotriphosphates

## Abstract

By taking the IR spectra of several cyclotriphosphates of a resolved structure, has subsequently shown that it is possible to characterize the P_3_O_9_ ring by its IR spectrum and, in some favorable cases, to make them Predicted symmetry of the cycle by examining the number, profile and position of the observed infrared bands in the symmetric valence vibration of the POP (νs POP) groups. He identified criteria for each type of symmetry and discussed, using concrete examples, the limits of the infrared method in determining the symmetry of the cycle (all the possible symmetries that a P_3_O_9_ cycle can have). Recently, at the Laboratory, studies have been undertaken by A. ABOUIMRANE et al. [1] for the calculation of the normal IR frequencies of the P_3_O_9_ cycle for the ideal and real symmetries: D_3h_, C_s_ and C_3_ (Tables 1,2 and 3).

Published by Elsevier Inc. This is an open access article under the CC BY license https://doi.org/10.1080/10426507.2017.1333507.

**Table:** Specifications Table

Subject area	*Chemistry*
More specific subject area	*Spectroscopy*
Type of data	*Table*
How data was acquired	*Infrared and Raman spectroscopy*
Data format	*analyzed, calculated*
Experimental factors	*These calculations were conducted using the semi-empirical method, Modified Neglect of Differential Overlap*
Experimental features	*The calculation of the frequencies was carried out first of all for the highest symmetry that the P*_*3*_*O*_*9*_*cycle can have, that of its molecular group D*_*3h*_*, then it was carried out for lower symmetries.*
Data source location	*Laboratory of Physical Chemistry of Materials LCPM, Faculty of Sciences Ben M'sik, B.P. 7955. Bd Cdt Driss El Harti. Hassan II University of Casablanca. Morocco*
Data accessibility	*With this article*
Related research article	*S. Zerraf, M. Belhabra, A.Kheireddine, R. Lamsatfi, M.Tridane, H. Moutaabbid,B. Baptiste, M. Moutaabbid, and S. Belaaouad, Reinvestigation of the crystal structure of barium cesium Cyclotriphosphate dihydrate and vibrational study, Phosphorus Sulfur Silicon Relat Elem., 192, 2017, 1286–1293*[2]*.*

**Table:** 

**Value of the data** • These data are useful for researchers working on spectral spectroscopy of cyclotriphosphates. • These data can be used to develop the spectral vibration of the cyclotriphosphate because they contain experimental vibrations and calculated vibrations. • The added value of these data is in the theoretical and experimental study of infrared and Raman frequencies in the different symmetric cyclotrophosphate, which contributes to the development of research in the spectral field.

## 1 Data

**Fig. 1:**
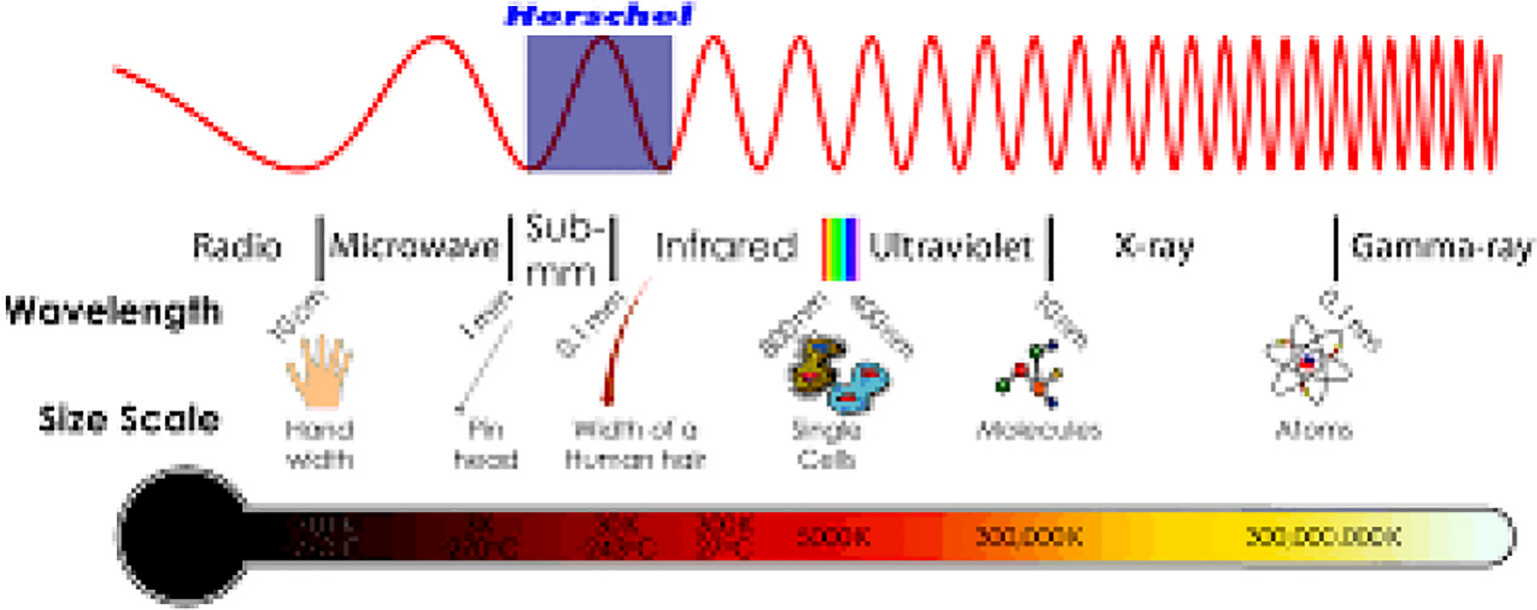
Infrared radiation.

**Fig. 2:**
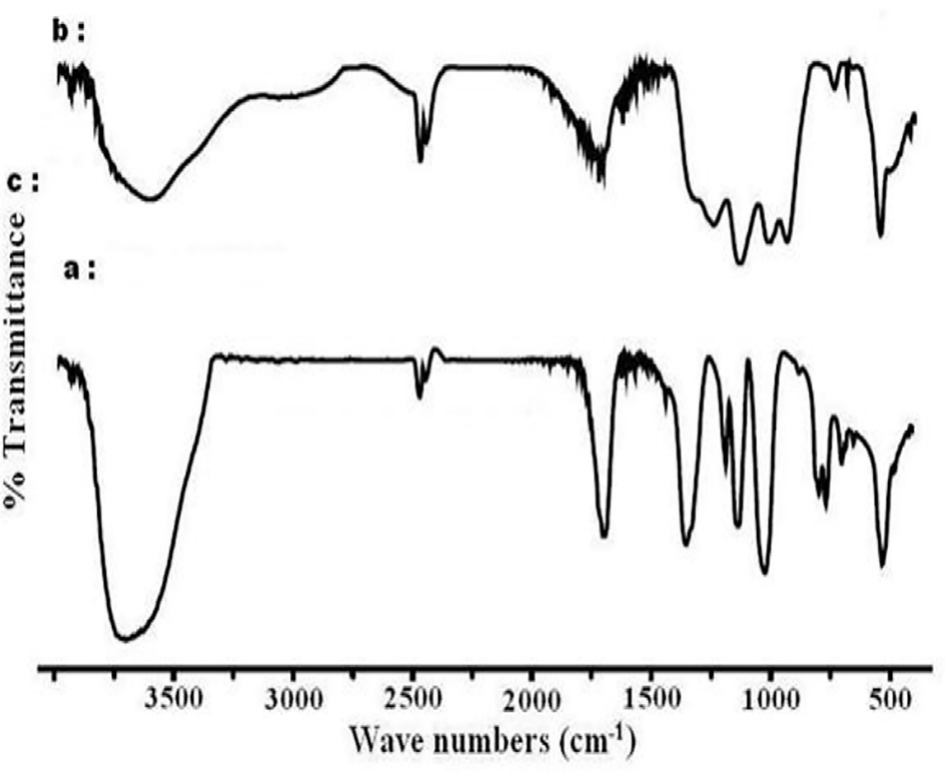
IR spectra of cyclotriphosphates. (a) CoK_4_(P_3_O_9_)_2_.7H_2_O, (b) NiK_4_(P_3_O_9_)_2_.7H_2_O.

**Fig. 3:**
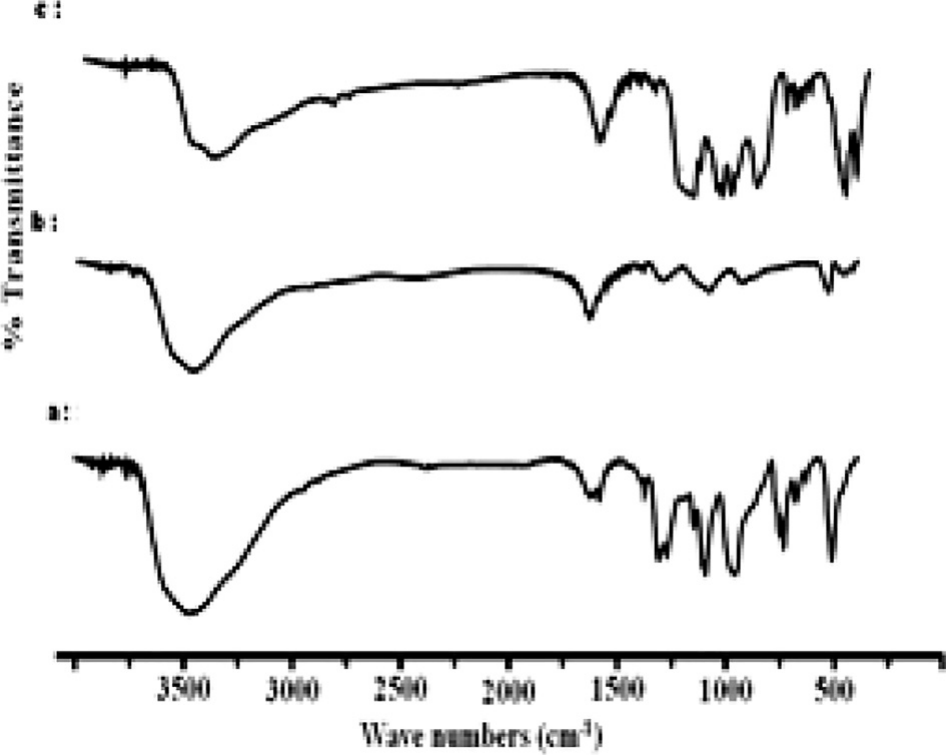
*IR spectra of cyclotriphosphates.* (a) SrNH_4_P_3_O_9_.3H2O, (b) SrRpP_3_O_9_.3H2O, (c) SrKP_3_O_9_.3H2O.

**Fig. 4:**
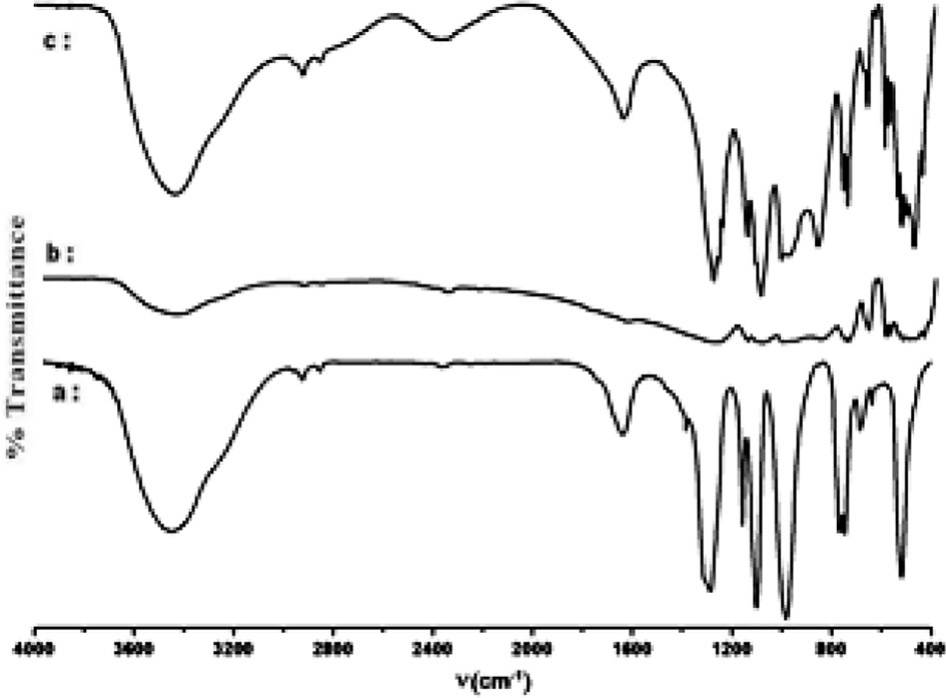
IR spectra of cyclotriphosphates. (a) ZnK_4_(P_3_O_9_)_2_.6H_2_O, (b) ZnRb_4_(P_3_O_9_)_2_.6H_2_O, *(c) NiRb*_*4*_*(P*_*3*_*O*_*9*_*)*_*2*_*.6H*_*2*_*O*.

The dataset shows how to determine different types of spectral vibration, as shown in Fig. 1. Table 1, Table 2, Table 3 refer to the frequencies to be calculated using different simulations in infrared and Raman spectroscopy. The comparison between the experimental and calculated vibration frequencies shows a total of 30 normal vibration patterns were identified for the isolated symmetry cycle D3h.The normal frequency calculation of the P3O9 cycle makes it possible to calculate the values of the internal vector component corresponding to the displacement of each atom of the cycle (see Fig. 2, Fig. 3, Fig. 4).

**Table 1:**
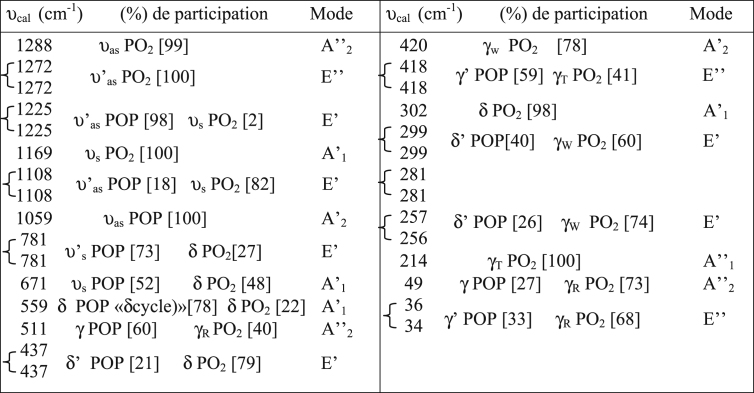
Calculated IR frequencies for symmetry D_3h_.

**Table 2:** Calculated IR frequencies for symmetry Cs.

υ_cal_ (cm^−1^)	(%) de participation		mode	υ_cal_ (cm^−1^)	(%) de participation		mode
1299	υ_as_ PO_2_ [98]		A′	427	δ′ POP [24]	δ′ PO_2_ [76]	A″
1280	υ_as_ PO_2_ [100]		A′	420	δ′ POP [44]	δ′ PO_2_ [56]	A′
1280	υ_as_ PO_2_ [100]		A″	415	γ_W_ PO_2_ [77]		A″
1200	υ_as_ POP [98]	υ_s_PO_2_ [2]	A″	305	δ POP [11]	δ PO_2_ [89]	A′
1188	υ_a_ POP [96]	υ_s_(PO_2_) [4]	A′	303	γ′ POP [16]	γ _T_ PO_2_ [84]	A″
1155	υ_s_ PO_2_ [98]		A′	297	δ′ POP [30]	γ _W_ PO_2_ [70]	A′
1099	υ_as_ POP [18]	υ_s_ PO_2_ [82]	A″	287	γ′ POP [14]	γ _T_ PO_2_ [86]	A′
1095	υ_as_ POP [21]	υ_s_PO_2_ [79]	A′	268	δ′ POP [33]	γ _W_ PO_2_ [67]	A″
1032	υ_as_ POP [94]		A″	255	δ′ POP [24]	γ _W_ PO_2_ [76]	A″
794	υ_s_ POP [77]	δ PO_2_ [23]	A″	252	δ′ POP [20]	γ_W_ PO_2_ [80]	A′
792	υ_s_ POP [76]	δ PO_2_ [24]	A′	214	γ _T_ PO_2_ [99]		A″
698	υ_s_ POP [64]	δ PO_2_ [36]	A′	104	γ POP [22]	γ _R_ PO_2_ [78]	A′
563	δ POP [71]	δ PO_2_ [29]	A′	89	γ′ POP [31]	γ _R_ PO_2_ [69]	A″
513	γ POP [63]	γ _R_ PO_2_ [37]	A′	59	γ′ POP [31]	γ_R_ PO_2_ [69]	A′
447	γ′ POP [62]	γ_T_ PO_2_ [38]	A″				
447	γ′ POP [49]	γ _T_ PO_2_ [51]	A′

**Table 3:**
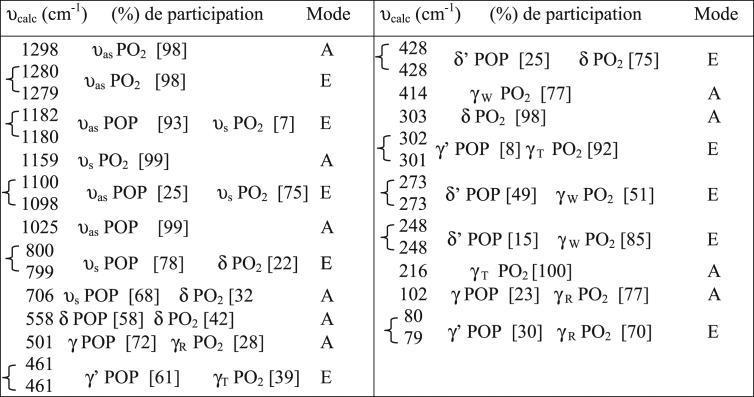
Calculated IR frequencies for symmetry C_3_.

For each frequency, the percentage of participation of the vibrations that contributed to it was specified. The percentages of the two groups, P-Oi-P and POe2 of the ring, were calculated from the successive isotopic substitutions 31P–33P, 16Oi-18Oi and 16Oe-18Oe. It has been assumed that internal oxygens are not involved in POe2 movements and that oxygens outside the cycle are not involved in POiP movements. The behavior of the eigenvectors, the displacement of the atoms with respect to their equilibrium position, and therefore of the relative movements at each normal frequency, with respect to the elements of symmetry of the group of the isolated P3O9 cycle, makes it possible to specify their symmetry and consequently the normal modes Corresponding. The assignment of the cycle frequencies is made without any a priori hypothesis and without vibrational spectra [1].

These allocations (Table 1, Table 2, Table 3) of the frequencies calculated for the corresponding modes for the symmetries D_3h_, C_s_ and C_3_ respectively were confirmed by the IR and Raman vibrational spectra of the compounds containing the P_3_O_9_ cycles of symmetry C_s_ (Table 5). This table shows how the normal modes change from the symmetry D_3h_ to the symmetry Cs of the isolated cycle. It shows the concordance between the values of the calculated frequencies and the experimental frequencies observed. Indeed, the IR spectra (Table 4) and those of Raman microspectrometry (Table 6) confirm the proposed assignments of both the valence frequencies and the deformation frequencies of the P_3_O_9_ cycle.

**Table 5:**
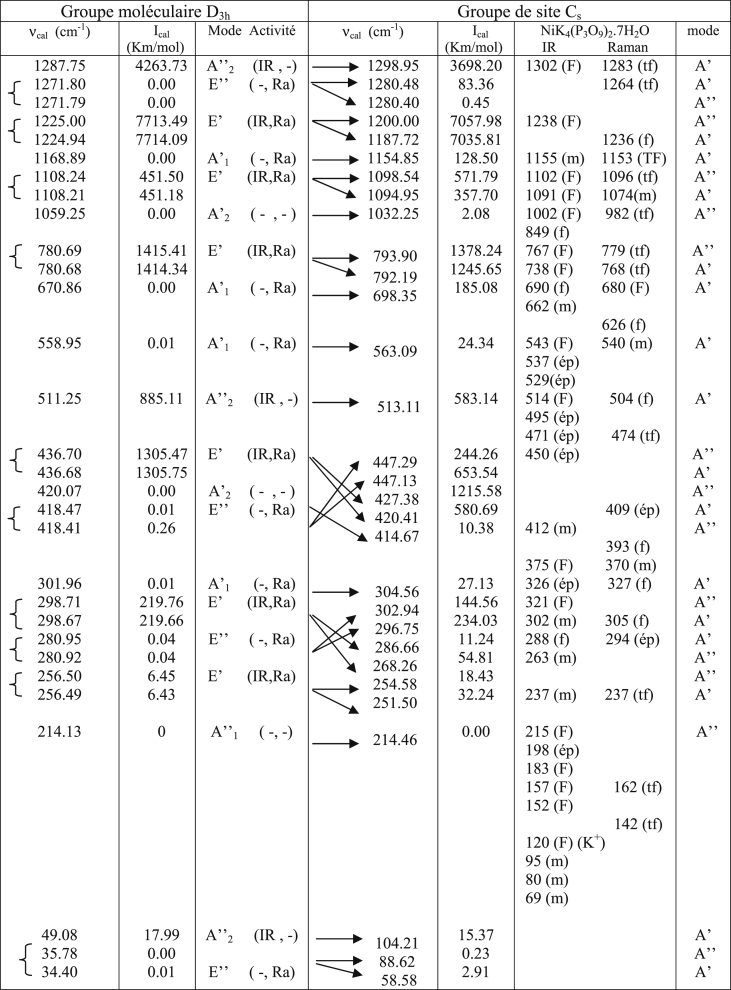
Assignment of the calculated frequencies to the corresponding modes for the Cs symmetry of the P_3_O_9_ cycle.

**Table 4:** IR and far IR frequencies (in cm^−1^) observed in cyclotriphosphates with a P_3_O_9_ cycle of symmetry C_s_: SrRbP_3_O_9_.3H2O (I), SrNH_4_P_3_O_9_.3H_2_O (II), SrKP_3_O_9_.3H_2_O (III),CoK_4_(P_3_O_9_)_2_.7H_2_O (IV), NiK_4_(P_3_O_9_)_2_.7H_2_O (V), ZnK_4_(P_3_O_9_)_2_.6H_2_O (VI), ZnRb_4_(P_3_O_9_)_2_.6H_2_O (VII) et NiRb_4_(P_3_O_9_)_2_.6H_2_O (VIII).

(I)	(II)	(III)	(IV)	(V)	(VI)	(VII)	(VIII)
1296 (F) 1255 (F)	1289 (F) 1265 (F) 1249(m)	1306 (F) 1274 (F)	1302 (F)	1302 (F)	1284 (F) 1255 (F)	1278 (F) 1267 (F)	1281 (F) 1267 (ép)
1203 (f)			1237 (F)	1238 (F)	1196 (f)	1196 (f)	1195 (f)
1157 (F)	1160 (F)	1162 (m)	1155(m)	1155 (m)	1155 (m)	1154 (m)	1159 (m)
1093 (F)	1094 (F)	1122 (F) 1097 (F)	1102 (F)	1102 (F) 1091 (F)	1096 (F)	1100 (F)	1096 (F)
1061 (f) 989 (F) 966 (F)	995 (ép) 974 (F)	1009 (F) 972 (F)	1002 (F)	1002 (F)	1031 (F) 1014 (F)	1021 (F)	1015 (F)
862 (f) 826 (f)	860 (ép)		861 (f)	849 (f)	879 (f)	881 (f)	923 (ép) 826 (f)
766 (F) 721 (f)	769 (F)	767 (F) 735 (F)	770 (ép) 744 (F)	767 (F) 738 (F)	779 (ép) 744 (F)	08 (m) 741 (F)	743 (F)
700 (f) 652 (f)	700 (F) 662 (f) 651 (m)	682 (m) 638 (f)	667 (f)	690 (f) 662 (m)		641 (m)	679 (ép) 641 (f)
606 (f)							
528 (F)	538 (F)	537 (F)	540 (m)	543 (F) 537 (ép) 529 (ép)	520 (ép)	548 (ép)	541 (ép)
517 (m)	516 (F)	512 (m) 510 (F)	521 (ép) 512 (F)	514 (F)	514 (F) 506 (ép)	514 (F)	504 (F)
498 (f) 457 (f)	457 (m) 387 (f) 374 (f) 364 (m) 336 (m) 322 (m) 312 (F) 289 (F) 215 (F) 191(F) (Sr^2+^) 183 (F) 161 (F) 146 (m) 117(F)(NH_4_^+^) 82 (m) 71 (F) 67 (ép) 61 (m)	452 (m) 384 (m) 366 (F) 335 (m) 323 (m) 310 (m) 283 (F) 218 (F) 205 (m) 192(F)(Sr^2+^) 172 (F) 125 (F) 109 (F)(K^+^) 105 (F) 77 (F) 65 (f)	493 (ép) 469 (m) 412 (f) 376 (m) 354 (f) 336 (m) 323 (m) 305 (f) 295 (ép) 261 (ép) 250 (m) 229 (f) 213(f)(Co^2+^) 174 (F) 154 (F) 139 (m) 130 (ép) 116(m)(K^+^) 110(m)(K^+^) 93 (m) 70 (m)	495 (ép) 471 (ép) 450 (ép) 412 (m) 375 (F) 326 (ép) 321 (F) 302 (m) 288 (f) 263 (m) 237 (m) 215(F)(Ni^2+^) 198 (ép) 183 (F) 157 (F) 152 (F) 120 (F)(K^+^) 95 (m) 80 (m) 69 (m)	486 (ép) 465 (m) 382 (f) 338 (m) 329 (ép) 314 (f) 301 (m) 273 (f) 236 (m) 205 (ép) 190(F)(Zn^2+^) 161 (m) 124 (F) 110 (ép)(K^+^) 92 (ép) 74 (f) 72 (f) 62 (ép)	464 (m) 400 (ép) 388 (f) 338 (m) 331 (ép) 310 (ép) 300 (m) 271 (f) 236 (m) 183(F)(Zn^2+^) 142 (ép) 127 (F) 95 (F)(Rb^+^) 75 (m) 60 (f) 55 (m)	464 (m) 396 (f) 341 (F) 307 (F) 274 (m) 256 (f) 235 (m) 210(F)(Ni^2+^) 179 (f) 150 (F) 100 (F)(Rb^+^) 95 (F)(Rb^+^) 72 (f)

**Table 6:** Distribution of the normal modes of vibration of the P_3_O_9_^3−^ ion in the isolated state of the various possible symmetries.

Groupe	Γvib (P_3_O_9_^3−^)	Activité		Coïncidence
Moléculaire		IR	Ra	
D_3h_	4 A′_1_ (Ra) + A″_2_+ 2 A′_2_ + 3 A″_2_ (IR) + 6 E’ (IR, Ra) + 4 E” (Ra)	9	14	6
*C_3h_	6 A’ (Ra) + 6 E’ (IR, Ra) + 4 A” + 4 E” (Ra)	10	16	6
*∼ C_3v_	7 A_1_ (IR, Ra) + 3 A_2_ + 10 E (IR, Ra)	17	17	7
*∼ C_2v_	10 A_1_ (IR, Ra) + 5 A_2_ (Ra) + 7 B_1_ (IR, Ra) + 8 B_2_ (IR, Ra)	25	30	25
*C_3_	10 A (IR, Ra) + 10 E (IR, Ra)	20	20	20
*C_2_	15 A (IR, Ra) + 15 B (IR, Ra)	30	30	30
*C_s_	17 A’ (IR, Ra) + 13 A” (IR, Ra)	30	30	30
C_s_	16 A’ (IR, Ra) + 14 A” (IR, Ra)	30	30	30
*C_1_	30 A (IR, Ra)	30	30	30

*: The currently known symmetries of the P_3_O_9_ ring.

(Table 7) gives the calculated IR frequencies for the symmetries D_3h_, C_s_ and C_3_ and specifies their variations with respect to those calculated for the highest symmetry D_3h_.

**Table 7:**
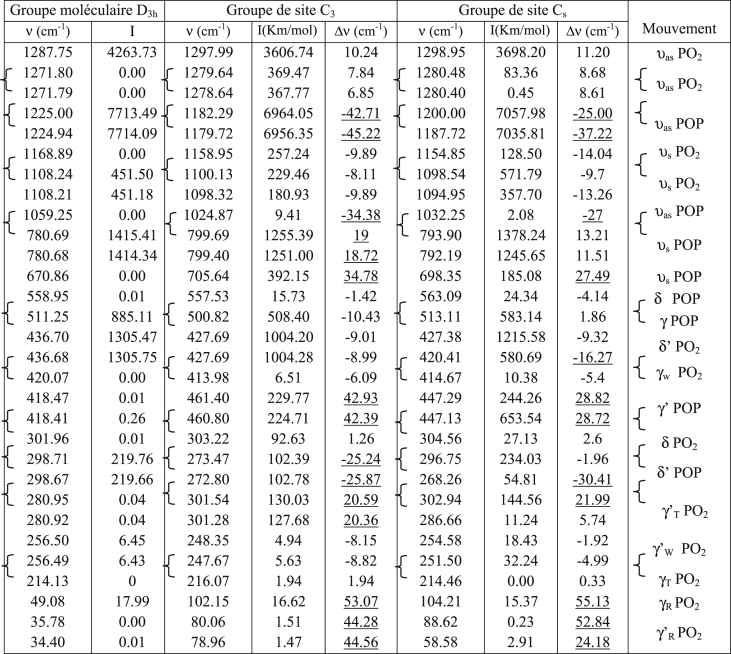
Calculated IR frequencies for the symmetries C_3_ and C_s_ and their variations with respect to those of the symmetry D_3h_.

## 2 Experimental design, materials and methods

These calculations were carried out using the semi-empirical method, Modified Neglect of Differential Overlap (MNDO) [2]. Thus, the calculation made it possible to obtain, for each of the normal frequencies of the P_3_O_9_ cycle, the values of the components of the eigenvectors corresponding to the displacements of each atom of the cycle.

For the calculated normal frequencies of the P_3_O_9_ cycle, the geometric variations of the elongations and angular deformations of the 12 P_3_O_9_ ring atoms corresponding to each were calculated. These movements made it possible to attribute the twelve fundamental valence frequencies, for which the variations of distances, P-Oe or P-Oi, are the most important at the 12 highest frequencies. Whereas for the other 18 vibrations of angular deformations the variations of the distances are zero or very small. On the basis of the atomic displacements, the valence frequencies and the deformation frequencies of the P_3_O_9_ cycle were distinguished and assigned.
